# Deletions of *NRXN1* (Neurexin-1) Predispose to a Wide Spectrum of Developmental Disorders

**DOI:** 10.1002/ajmg.b.31063

**Published:** 2010-04-07

**Authors:** Michael SL Ching, Yiping Shen, Wen-Hann Tan, Shafali S Jeste, Eric M Morrow, Xiaoli Chen, Nahit M Mukaddes, Seung-Yun Yoo, Ellen Hanson, Rachel Hundley, Christina Austin, Ronald E Becker, Gerard T Berry, Katherine Driscoll, Elizabeth C Engle, Sandra Friedman, James F Gusella, Fuki M Hisama, Mira B Irons, Tina Lafiosca, Elaine LeClair, David T Miller, Michael Neessen, Jonathan D Picker, Leonard Rappaport, Cynthia M Rooney, Dean P Sarco, Joan M Stoler, Christopher A Walsh, Robert R Wolff, Ting Zhang, Ramzi H Nasir, Bai-Lin Wu, on behalf of the Children's Hospital Boston Genotype Phenotype Study Group

**Affiliations:** 1 Division of Developmental Medicine, Children's Hospital BostonBoston, Massachusetts; 2 Harvard Medical SchoolBoston, Massachusetts; 3 Center for Human Genetic Research, Massachusetts General HospitalBoston, Massachusetts; 4 Division of Genetics, Children's Hospital BostonBoston, Massachusetts; 5 Department of Neurology, Children's Hospital BostonBoston, Massachusetts; 6 Department of Molecular Biology, Cell Biology and Biochemistry, Brown UniversityProvidence, Rhode Island; 7 Department of Laboratory Medicine, Children's Hospital BostonBoston, Massachusetts; 8 Department of Molecular Immunology, Capital Institute of PediatricsBeijing, China; 9 Istanbul Faculty of Medicine, Department of Child Psychiatry, Istanbul UniversityIstanbul, Turkey; 10 Children's Hospital Boston, Howard Hughes Medical InstituteBoston, Massachusetts; 11 Manton Center for Orphan Disease Research, Children's Hospital BostonBoston, Massachusetts; 12 Department of Ophthalmology, Children's Hospital BostonBoston, Massachusetts; 13 Department of Genetics, Harvard Medical SchoolBoston, Massachusetts; 14 Howard Hughes Medical Institute, Beth Israel Deaconess Medical CenterBoston, Massachusetts; 15 Children's Hospital and Institutes of Biomedical Science, Fudan UniversityShanghai, China

**Keywords:** *NRXN1* (neurexin-1), developmental disorders, array CGH, *NRXN1* exonic deletions, CNV

## Abstract

Research has implicated mutations in the gene for neurexin-1 (*NRXN1*) in a variety of conditions including autism, schizophrenia, and nicotine dependence. To our knowledge, there have been no published reports describing the breadth of the phenotype associated with mutations in *NRXN1*. We present a medical record review of subjects with deletions involving exonic sequences of *NRXN1*. We ascertained cases from 3,540 individuals referred clinically for comparative genomic hybridization testing from March 2007 to January 2009. Twelve subjects were identified with exonic deletions. The phenotype of individuals with *NRXN1* deletion is variable and includes autism spectrum disorders, mental retardation, language delays, and hypotonia. There was a statistically significant increase in *NRXN1* deletion in our clinical sample compared to control populations described in the literature (*P* = 8.9 × 10^−7^). Three additional subjects with *NRXN1* deletions and autism were identified through the Homozygosity Mapping Collaborative for Autism, and this deletion segregated with the phenotype. Our study indicates that deletions of *NRXN1* predispose to a wide spectrum of developmental disorders. © 2010 Wiley-Liss, Inc.

## INTRODUCTION

Neurexins are a group of highly polymorphic cell surface proteins involved in synapse formation and signaling [Ushkaryov et al., 1992; Missler and Sudhof, 1998; Missler et al., 2003; Graf et al., 2004; Nam and Chen, 2005]. There are three human neurexin genes (*NRXN1*, *NRXN2*, and *NRXN3*), each of which has two independent promoters resulting in an α and a β neurexin for each gene [Ushkaryov et al., 1992; Ichtchenko et al., 1996]. Multiple alternative splicing leads to the possibility of greater than a thousand distinct neurexin isoforms [Ullrich et al., 1995]. Their expression is believed to be spatially and temporally regulated throughout development [Puschel and Betz, 1995; Zeng et al., 2006].

### Structure and Function of *NRXN1*

*NRXN1*, located on chromosome 2p16.3, is one of the largest known human genes (1.1 Mb with 24 exons) [Tabuchi and Sudhof, 2002]. It is subject to relatively frequent disruption including missense changes, translocation, whole gene deletion, and intragenic copy number alterations [Feng et al., 2006; Szatmari et al., 2007; International Schizophrenia Consortium, 2008; Kim et al., 2008; Kirov et al., 2008; Marshall et al., 2008; Morrow et al., 2008; Yan et al., 2008; Zahir et al., 2008; Glessner et al., 2009; Rujescu et al., 2009].

**FIG. 1:**
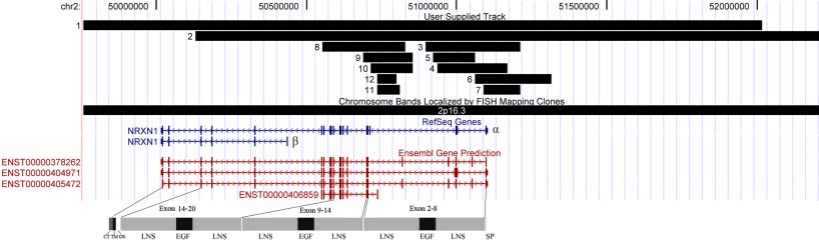
Illustrates the size and range of the 12 deletion CNVs in relation to the exons and protein domains of NRXN1-α and -β in the UCSC Genome Browser (http://genome.ucsc.edu) [Kent et al., 2002]. The top track shows the genomic position and size of the 12 deletion CNVs. The middle tracks show the gene annotations in RefSeq and Ensembl. The Refseq Genes show the α and β isoforms of the NRXN1 gene; the Ensembl gene prediction shows several other minor isoforms of the NRXN1 gene. The bottom panel shows the protein domains of the NRXN1-α gene product. SP, signal peptide; LNS, laminin/neurexin/sex hormone-binding globulin domain; EGF, epithelium growth factor like domain; OS, *O*-glycosylation sequence; TM, transmembrane domain; CT, cytoplasmic tail. [Color figure can be viewed in the online issue, which is available at http://www.interscience.wiley.com.]

The longer transcript, *NRXN1*-α, encodes an N-terminal signal peptide with three repeats of two laminin/neurexin/sex hormone-binding globulin (LNS) domains separated by an EGF-like sequence (Fig. 1). Following these repeats, there is an *O*-glycosylation sequence, a transmembrane domain, and a cytoplasmic tail of 55 amino acids.

Neurexin-1-α has been shown to interact with certain neuroligin isoforms and neurexin-binding proteins known as neurexophilins. This presynaptic molecule is also required for calcium-triggered neurotransmitter release and the function of voltage-gated calcium channels in the synapses of the brainstem and neocortex [Missler et al., 2003; Zhang et al., 2005; Dudanova et al., 2006]. Mouse knockouts of all three α-neurexin genes do not demonstrate major abnormalities of axonal pathfinding during development [Dudanova et al., 2007], although synaptic function is severely impaired. Mice with knockouts of individual α-neurexin genes have modestly decreased postnatal viability, while double knockout mice have greatly decreased postnatal survival. Triple knockout mice do not survive past the first day of life [Missler et al., 2003].

Neurexin-1-β is much shorter than Neurexin-1-α, as five of the six LNS domains and the intervening EGF sequences are replaced with a short β-neurexin-specific sequence (Fig. 1) [Missler and Sudhof, 1998]. Neurexin-1-β has been shown to interact with the postsynaptic neuroligin family of cell adhesion molecules and dystroglycans [Ichtchenko et al., 1995; Sugita et al., 2001; Arac et al., 2007; Comoletti et al., 2007; Chen et al., 2008]. No mouse models with knockouts of *NRXN1*-β, alone or in combination with *NRXN1*-α, have yet been analyzed [Sudhof, 2008]. For each of Neurexin-1-α and Neurexin-1-β, multiple protein coding isoforms of *NRXN1* have been identified, whose structure and functions are not well understood.

### *NRXN1* Mutations in Humans

There is increasing evidence that *NRXN1* disruptions [Kim et al., 2008], point mutations [Feng et al., 2006; Yan et al., 2008], and deletions [Glessner et al., 2009; Marshall et al., 2008; Morrow et al., 2008; Szatmari et al., 2007] are associated with autism spectrum disorders. *NRXN1* has also been found to be associated with autism in a large genome-wide single nucleotide polymorphism association study [Wang et al., 2009].

*NRXN1* deletions have also been associated with a variety of other conditions including schizophrenia [International Schizophrenia Consortium, 2008; Kirov et al., 2008; Vrijenhoek et al., 2008; Walsh et al., 2008; Need et al., 2009; Rujescu et al., 2009], nicotine dependence [Bierut et al., 2007; Nussbaum et al., 2008], and other physical manifestations such as vertebral anomalies [Zahir et al., 2008].

Prior reports of abnormalities in *NRXN1* have focused on populations with specific diagnoses (e.g., autism, schizophrenia). However, the clinical significance of copy number variants (CNV), such as deletion involving one or more exons of *NRXN1*, and the range of phenotypic manifestations of subjects with *NRXN1* deletion CNV remains unclear. We describe here a group of subjects with *NRXN1* deletions who demonstrate a wide range of physical and developmental phenotypes.

## MATERIALS AND METHODS

### Clinical Cohort Record Review

From March 2007 to January 2009, a total of 3,540 subjects at Children's Hospital Boston were evaluated for genomic imbalance (deletion and duplication) using the Agilent 244K human genome oligonucleotide comparative genomic hybridization (CGH) microarrays (G4411B, Agilent Technologies, Palo Alto, CA) according to the manufacturer's instructions [Oligonucleotide Array-Based CGH for Genomic DNA Analysis protocol version 3 (Agilent Technologies)]. The majority of the referrals were for clinical features of developmental disorders (developmental delay, autism spectrum disorders, mental retardation) or multiple congenital malformations as determined by specialists in Clinical Genetics, Neurology, and Developmental Medicine.

One hundred thirty probes cover the 1.12 Mb region of the *NRXN1* gene on the Agilent 244K CGH array. The average interprobe space within the *NRXN1* gene is 8.6 kb. This permits the reliable detection of small intragenic deletions down to 43 kb in size. Images were captured by Agilent scanner and quantified using Feature Extraction software v9.0 (Agilent Technologies). CGH Analytics Software v3.4 (Agilent Technologies) was subsequently used for data normalization, quality evaluation and data visualization. Copy number aberration was indicated using the Aberration Detection Method 2 (ADM-2) algorithm. Deletions involving five or more consecutive probes were considered as true CNV.

For two larger deletions, fluorescent in situ hybridization (FISH) testing using probe RP11-800C7 was carried out for deletion confirmation and parental testing. The smaller deletions were confirmed by PCR-based breakpoint mapping methods. The primers used for each case are listed in the Supplementary Material.

Subjects with deletions involving exonic sequence of *NRXN1* were included in our review. Two developmental behavioral pediatricians (RHN, MSLC), a clinical geneticist (WHT), and a pediatric neurologist (SSJ) reviewed each of the medical records. The clinical history, physical examination, laboratory data, and radiological reports of each subject were reviewed.

### Additional Report of Cases With *NRXN1* Deletion and Autism

Cases with exonic and intragenic *NRXN1* deletions were also contributed from the Homozygosity Mapping Collaborative for Autism (HMCA) which utilized the Affymetrix GeneChip Human Mapping 500K Array Set using CNV detection methods previously described [Morrow et al., 2008].

This work was approved by the Institutional Review Boards at the corresponding hospitals.

## RESULTS

### Clinical Cohort Record Review

We identified 12 subjects through Children's Hospital Boston with deletions involving exonic sequences of *NRXN1* (Table I and Fig. 1). The deletions reported here range from 65 kb to 5 Mb and most of these cases are predicted to affect the initial structural domains of the protein (Fig. 1).

**TABLE I:** Deletions Within *NRXN1* in Our Sample

Patient	Deletion location (hg18 build)	Size of deletion (kb)	Inheritance	Exons–introns deleted	Other genetic tests and results (additional imbalance)	Indication for testing	Confirmation method
1	46,938,685–52,015,885	5,077	Maternal FISH normal; paternal study unavailable	All	Karyotyping and Fragile X test: normal (contiguous deletion including FSHR, LHCGR, STN1)	Moderate mental retardation	FISH
2	50,128,256–54,050,713	3,923	De novo	All except the last two exons	None	Global developmental delays, suspected autism	FISH
3	50,897,002–51,212,385	315	Paternal	Exon 1–5; partial intron 5	Karyotyping and chromosome 15 methylation: normal	Gross motor delay, hypotonia	PCR
4	50,936,914–51,167,934	231	Paternal	Exon 1–5; partial intron 5	Karyotyping, fragile X test, *SALL1*, and *CHD7* mutation test: normal	PDD-NOS, hypotonia	PCR
5	50,920,082–51,059,469	139	De novo	Exon 3, 4, 5; partial introns 2, 5	None	VACTERL	Not done
6	51,059,410–51,316,396	257	Maternal	Exon 1, 2; partial intron 2	Karyotyping and fragile X test: normal	PDD-NOS, motor coordination delays	PCR
7	51,090,504–51,212,385	122	Paternal	Exon 1–3; partial intron 3	Karyotyping, Fragile X test, and *PTEN* mutation test: normal	Autism, moderate mental retardation	PCR
8	50,522,892–50,827,767	305	De novo	Exon 6–17; partial introns 5, 17^a^	Fragile X test: normal (deletion at 3p24.3 from 21492764 to 21806824, maternally inherited)	Mild mental retardation	PCR
9	50,689,280–50,853,329	164	Unknown (foster family)	Exon 6–8; partial introns 5, 8^a^	Karyotyping: normal	Language delay, prenatal substance exposure	PCR
10	50,714,297–50,853,329	139	De novo	Intron 5^a^	Karyotyping and fragile X test: normal	PDD-NOS	PCR
11	50,735,499–50,811,018	76	Maternal	Intron 5^a^	Karyotyping, *PTEN*, and *NSD1* mutation tests: normal (duplications at 5p13.2 from 37241141 to 37758854, paternally inherited; at 15q26.3 from 98059710 to 98842423, maternally inherited; at 17p11.2 from 21147675 to 21442522 maternally inherited)	Hypotonia, muscle weakness, large birth weight	PCR
12	50,735,499–50,801,233	66	Maternal	Intron 5^a^	None	Poor weight gain, mild craniofacial dysmorphism	PCR

*FSHR*, follicle-stimulating hormone receptor; *LHCGR*, luteinizing hormone/choriogonadotropin receptor; *STN1*, *Stoned B*-like factor; PDD-NOS, pervasive developmental disorder, not otherwise specified; VACTERL, vertebral anomalies, anal atresia, cardiac malformations, tracheoesophageal fistula, renal anomalies, and limb anomalies; *SALL1*, sal-like 1 (Drosophila); *CHD7*, chromodomain helicase DNA-binding protein 7; *PTEN*, phosphatase and tensin homolog; *NSD1*, nuclear receptor-binding SET domain protein 1.

a Deletions of intron 5 in these patients involve an exon of a minor isoform of *NRXN1*.

Of these 12 deletions, 4 were de novo CNV not identified in either parent, 3 were maternally inherited, 3 were paternally inherited, and the parental samples for 1 (subject 9) were not available. In subjects 1, paternal samples were not available but the deletion was not identified in maternal testing.

In subjects 1–9, the deletions involved at least two exons of *NRXN1*-α, while in subjects 10–12, the deletions involved only an exon of a minor expressed *NRXN1* isoform. The genomic imbalances involving *NRXN1* are summarized in Table I and the clinical manifestations are summarized in Tables II and III. Further clinical data are available in the Supplementary Material.

**TABLE II:** Neurological and Developmental Characteristics

Subject	Sex	Age at ascertainment	Autism spectrum disorder	Cognitive-developmental findings	Language delay	Motor involvement	History of seizures/EEG results	MRI-brain	Behavioral features
1	M	16 y	No	MR; SB5: FSIQ 44; VIQ 44; NVIQ 48; (CA 14 y)	Expressive and receptive	Walked at 18 months	History of seizures; abnormal EEG	Normal	Inattention, impulsivity, hyperactivity
2	M	2 y	Autism suspected, no formal evaluation available	Global developmental delays	Expressive and receptive	Not documented	Not documented	Not performed or not documented	Not documented
3	F	10 mo	Not suspected	No concerns reported. Testing not documented	No	Mild gross motor delay, hypotonia	None	Not performed or not documented	Not documented
4	M	4 y	PDD-NOS (ADOS)	WPPSI-III VIQ 77, PIQ 98 (CA 4 y)	Expressive	Hypotonia	EEG Normal	Not performed or not documented	Attention concerns
5	F	6 y	No	No concerns reported. Testing not documented	6 month receptive delay	Normal	Not documented	Not performed or not documented	Not documented
6	F	7 y	PDD-NOS (ADOS)	Bayley II mental scale 91, 29 mo (CA 31 mo)	Expressive	Motor coordination disorder	None	Not performed or not documented	Not documented
7	M	14 y	Autism (ADOS)	MR: SB5: FSIQ 47; VIQ 46; NVIQ 53	Expressive and receptive	Normal	EEG normal	Not performed or not documented	Hyperactivity
8	F	11 y	No	MR: WISC-IV: VCI 67, PRI 63, WMI 59, PSI 75, FSIQ 58 (CA 11 y)	Expressive and receptive	Normal	None	Normal	Inattention, fidgety, disorganized
9	F	4 y	No	Academic delays reported. Testing not documented [Correction made here after initial online publication: Findings for subject 9 and 11 were inadvertently switched in the original online version]	Expressive and receptive	Hypotonia	None	Normal	Impulsivity and inattention
10	M	2 y	PDD-NOS (ADOS)	Bayley III cognitive score 95 (average)	Expressive and receptive	Normal	Not documented	Not performed or not documented	Not documented
11	M	8 y	No	No concerns reported. Testing not documented [Correction made here after initial online publication: Findings for subject 9 and 11 were inadvertently switched in the original online version]	No	Proximal and distal weakness, hypotonia	None	Not performed or not documented	Not documented
12	F	19 mo	Not documented	Not documented	Not documented	Normal	None	Not performed or not documented	Not documented

ADOS, autism diagnostic observation schedule; Bayley II, Bayley Scales of Infant Development, second edition; Bayley III, Bayley scales of infant and toddler development, third edition; CA, chronological age at testing; MR, mental retardation; SB5, Stanford-Binet intelligence scales, fifth edition; FSIQ, full scale IQ; VIQ, verbal IQ; NVIQ, non verbal IQ; PIQ, performance IQ; WPPSI-III, Wechsler preschool and primary scale of intelligence, third edition; WISC-IV, Wechsler intelligence scale for children, fourth edition; VCI, verbal comprehension index, PRI, perceptual reasoning index; WMI, working memory index; PSI, processing speed index; y, years; mo, months.

**TABLE III:** Relevant Physical Characteristics

Subject	Dysmorphic features	Vertebral/skeletal	Cardiac	Skin
1	None	Not documented	Normal	Not documented
2	Frontal bossing	History of plagiocephaly	Resolved heart murmur	Hemangioma on neck
3	Epicanthal folds; hypertelorism smaller bifrontal region	Prominent coronal sutures; feet: high arches and somewhat small length	Normal	Lighter than parents
4	Down-slanting palpebral fissures; anteverted nares; mild retrognathia, pointed chin	Not documented	Normal	Normal
5	None	Curved 2nd toes, incomplete fusion of ring of first cervical vertebra	Narrowed aortic arch, 2 VSDs	Not documented
6	None	Bilateral hip dysplasia	Prolonged QTc (457 msec)	Hemangioma on neck
7	Slightly deep set eyes, large ears	Normal	Normal	Normal
8	Long face, malar hypoplasia, prominent tubular nose with pointed nasal tip, hypoplastic alae nase, long flat philtrum, thin vermilion, prominent chin, long slender fingers, thin toes	Not documented	Normal	Normal
9	Low nasal bridge, small jaw, very smooth philtrum. Slightly flat mid-face and prominent cheeks	Mild clinodactyly and uneven digit lengths	Normal	Not documented
10	Dolichocephaly (32-week premature infant)	Not documented	Normal	Hemangioma on back
11	None	Chest-right mild Poland anomaly	Normal	Eczema
12	Relative macrocephaly (head circumference 90%), cupping of left ear, frontal bossing	Open anterior fontanelle at 19 months	Small muscular VSD, fenestration in atrial septum, small PDA	Not documented

VSD, ventricular septal defect; PDA, patent ductus arteriosus; QTc, corrected QT interval (normal <440 msec).

Detailed clinical records were available from geneticists in 9 out of 12 subjects, developmental-behavioral pediatricians in 6/12, psychologists in 6/12, and neurologists in 4/12. Four of the 12 subjects (4, 6, 7, and 10) were diagnosed with autism spectrum disorders; in each positive case, this diagnosis was supported by the Autism Diagnostic Observation Schedule. Another subject (2) was suspected of having autism but the evaluation was not available for review; he also had global developmental delays. Two subjects had mental retardation without a diagnosis of an autism spectrum disorder (1 and 8). Subject 1, in addition, had absence seizures and an EEG consistent with a primary generalized epilepsy. One subject (3) was too young to ascertain for an autism spectrum disorder or cognitive delays. Nine subjects had clinical documentation of expressive or receptive language delays.

Mild dysmorphic features were present in seven subjects (2, 3, 4, 7, 8, 9, and 12); three subjects had hemangiomas (2, 6, and 10). Hypotonia was present in four subjects (3, 4, 9, and 11). Two subjects (5 and 12) had ventricular septal defects.

Medical record review also revealed the following characteristics in the six parents from whom the NRXN1 deletion was inherited. Subject 4, who had pervasive developmental disorder, not otherwise specified (PDD-NOS) and hypotonia, inherited his deletion from his father who is also reported to be socially awkward. Subject 6, who had PDD-NOS and coordination issues, inherited the deletion from a mother with a history of language delay and social skill difficulties. Subject 11, who has hypotonia, weakness, and Poland anomaly, inherited the deletion from a mother who has a history of joint hypermobility, osteoarthritis, mitral valve prolapse, severe migraines, and severe breast asymmetry. The father of subject 3 (hypotonia, gross motor delay), the father of subject 7 (autism, mental retardation) and the mother of subject 12 (poor weight gain, craniofacial dysmorphism) are reported to be healthy without developmental or medical concerns.

### Additional Report of Cases With *NRXN1* Deletion and Autism

**FIG. 2:**
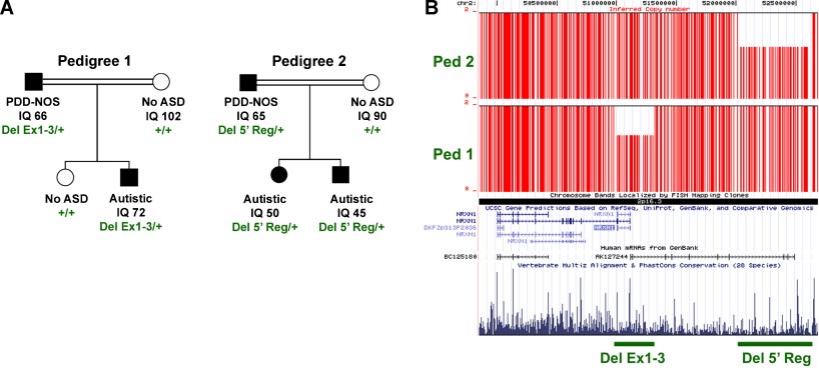
A: NRXN1-α deletions segregate with autism spectrum disorder (ASD) and mild mental retardation. Pedigree 1 shows co-segregation of a hemizygous CNV between rs17041500 and rs17512199 which deletes the first three coding exons (Del Ex1-3) of NRXN1-α. The CNV is carried by all subjects with ASD and diminished intelligence quotient (IQ), but not by a typically developing sibling. Pedigree 2 shows co-segregation of a hemizygous CNV which deletes likely regulatory, genomic DNA upstream (Del 5′Reg) of NRXN1-α. PDD-NOS, pervasive development disorder, not otherwise specified. +, wild-type, non-deleted DNA. **B**: Mapping of inferred CN data SNP-by-SNP on the UCSC genome browser demonstrates the extent across the NRXN1 locus. Vertical red lines indicate each SNP with copy number of 1 or 2. Horizontal green lines demarcate the extent of each deletion. Alignment of annotated genes in the RefSeq database are shown as well as a representation of vertebrate conservation using multiz and related tools in the UCSC/Penn State Bioinformatics comparative genomic alignment pipeline. Of note, Del 5′Reg deletes the last four exons of an uncharacterized, spliced mRNA AK127244 that is expressed in brain. The gene is transcribed in the opposite direction as NRXN1-α yet the transcription start site is within 3.5 kb suggesting that this mRNA may be transcribed coordinately with NRXN1-α. [Color figure can be viewed in the online issue, which is available at http://www.interscience.wiley.com.]

In addition to the Children's Hospital Boston cases, we report here three cases from two families ascertained through the HMCA [Morrow et al., 2008]. The *NRXN1* deletions in each were discovered to segregate with IQ below 70 in these pedigrees (Fig. 2). All three affected children were carriers and unaffected children were not. The deletions were inherited from fathers who were found to have ASD symptoms and IQ between 60 and 70, while non-carrier mothers were not on the autism spectrum and with IQs in the normal range. The deletion for the subject in the first family is exonic and intragenic, while the deletion for the two siblings in the second family is upstream and may affect gene expression. Further investigation is necessary to substantiate this as a functional deletion, even though it segregates with disease.

### Significance Test

To establish the relevance of these CNV, we compared the frequency of deletions involving *NRXN1*-α exons in our Children's Hospital Boston population, in whom CGH testing was considered to be clinically indicated, to the frequency of similar deletions detected by array genomic profiling of equivalent resolution in normal populations. Itsara et al. 2009 detected three deletions involving *NRXN1*-α exons in 2,493 normal individuals. The International Schizophrenia Consortium 2008 reported two exonic deletion cases in 3,181 normal controls. Another large-scale schizophrenia study identified five deletion cases among 33,746 normal controls [Rujescu et al., 2009]. Recently, Glessner et al. 2009 reported no deletion CNV involving *NRXN1*-α among 1,409 Autism Case–Control (ACC) control samples and 1,110 Autism Genetic Resource Exchange (AGRE) controls. Collectively, the frequency of exonic deletion of *NRXN1*-α in control populations is 10/51,939 (0.019%); this differs significantly from the frequency of exonic deletion CNV we observed in our clinically referred population (9/3,540) (0.25%; *P* = 8.9 × 10^−7^, two-tailed Fisher's exact test). There are no available data on the frequency of minor isoform exonic deletions in control populations and thus these subjects (n = 3) were excluded from the significance test.

## DISCUSSION

The recent recognition of genomic imbalance in many chromosomal regions that are associated with autism, mental retardation, and schizophrenia is due to the increasing use of whole genome high-resolution array CGH in the evaluation of individuals with these disorders. Our clinical subjects with *NRXN1* deletion were ascertained through a patient population presenting with a broad range of referring diagnoses.

Through a careful review of medical records, we identified in our subjects a number of clinical features that had not been previously associated with *NRXN1* deletions. These include language delays, mental retardation without autism, hypotonia, and hemangiomas.

In addition, two of our subjects (5 and 12) had ventricular septal defects. Interestingly, the human cDNA homologous to rat *NRXN1*-α has been isolated in both brain and heart tissues suggesting a potential role for Neurexin-1 in both brain and heart development [Nagase et al., 1998]. One of these subjects (5) also had evidence of multiple congenital anomalies including vertebral anomalies in the form of a VACTERL association. Vertebral anomalies have also been reported in one other case in the literature [Zahir et al., 2008].

A previous report showed the presence of a seizure disorder in two unrelated individuals sharing the same missense variant in exon 1 of *NRXN1*-β [Feng et al., 2006]. In our cohort, only one subject had a seizure disorder (subject 1), although his 5 Mb deletion encompassed the entire *NRXN1* gene as well as the genes for follicle-stimulating hormone receptor (*FSHR*), luteinizing hormone/choriogonadotropin receptor (*LHCGR*), and *Stoned B*-like factor (*STN1*). To our knowledge, none of these genes has been associated with seizures or mental retardation in the literature.

Although we cannot be certain that these features are a direct consequence of *NRXN1* deletion, our observations suggest that the phenotypic characteristics of *NRXN1* deletion may be wider than previously reported.

The mutations we have observed in our clinical cohort are primarily in *NRXN1*-α. Subjects with small deletions (under 3 Mb) clustered into two groups (Fig. 1). One group (subjects 3–7) had deletions involving part of the initial LNS and EGF domains-encoding regions of *NRXN1*-α. Of these five individuals, three had autism spectrum disorders. One additional case from the HMCA was also found to have a deletion in this region, which is similar to the deletion in subject 7 from the clinically referred cohort.

A second group (subjects 8–12) had deletions that clustered around a region further from the α promotor of the gene (Fig. 1). All five of these subjects' deletions encompassed an exon of an isoform whose function is not well understood. Furthermore, while two subjects (8 and 9) had deletions involving other exons of *NRXN1*-α as well as this minor isoform, three subjects' deletions (10–12) contain only the exon of this minor isoform. This minor isoform is an Ensembl annotated transcript, named ENST00000406859 (Fig. 1). It contains 13 exons with 2,590 bp transcription and 856 residues of translation length. The coded protein (ENSP00000385681) consists of one LNS and EGF domain. Its function is currently unknown.

One such subject (10) with a de novo deletion in this region has been diagnosed with PDD-NOS, suggesting potential clinical relevance for this isoform. This deletion in intron 5 has not to our knowledge been previously reported as being associated with abnormal development.

Neurexin-1-β mutations were less common. Two of the subjects in our cohort had large deletions encompassing exons for *NRXN1*-α and -β. Missense variants in *NRXN1*-β (R8P, L13F, S14L, and T40S) have previously been identified in individuals with autism [Feng et al., 2006; Kim et al., 2008]. Relatives of these individuals with autism who shared these missense mutations demonstrated some degree of learning or behavioral issues but did not appear to meet full autism spectrum disorder criteria [Feng et al., 2006; Kim et al., 2008]. This is consistent with our findings of a mixed phenotype associated with deletions in this region ranging from autism spectrum disorders to hypotonia with carrier relatives who are not as affected.

In addition to their *NRXN1* deletions, subjects 8 and 11 had additional genomic imbalances as described in Table I. These genomic imbalances were all inherited from unaffected parents. The two duplications on 15q26.3 and 17p11.2 in subject 11 overlap with known benign CNVs and are unlikely contributory factors to the patient's condition. The duplication at 5p13.2 in subject 11 and deletion at 3p24.3 in subject 8 are not previously reported CNV but contain no known genes associated with developmental disorders, thus are considered as CNV of unknown significance. Nevertheless, it is unclear whether these CNVs modified the observed phenotype.

### *NRXN1* and Synapse Function

Prior studies have functionally linked other molecules that are associated with *NRXN1* to a range of neuropsychiatric disorders including autism. These include neuroligins 3 and 4 (*NLGN3*, *NLGN4*) and *SH3* and multiple ankyrin repeat domains 3 (*SHANK3*) [Jamain et al., 2002; Laumonnier et al., 2004; Durand et al., 2007; Moessner et al., 2007; Lawson-Yuen et al., 2008]. In addition, *CNTNAP2* (contactin associated protein-like 2) [Alarcon et al., 2008; Arking et al., 2008; Bakkaloglu et al., 2008] and cadherin 10 (*CDH10*) and 9 (*CDH9*) have been also associated with autism spectrum disorders [Wang et al., 2009]. Our finding that *NRXN1* is also associated with autism and developmental disorders adds further evidence to the importance of this molecular family to the development of neurodevelopmental disorders.

The function of *NRXN1* in facilitating synaptic transmission suggests that mutations in this gene may predispose to a neurologic disconnection syndrome. Long-range disconnections between neural networks have been hypothesized to be causative in some populations with autism [Barnea-Goraly et al., 2004; Frith, 2004; Just et al., 2004; Geschwind and Levitt, 2007]. The effects of *NRXN1* on language development and hypotonia may likewise be related to long-range connectivity within the brain.

### Phenotypic Variation

Phenotypic variations may reflect the highly pleiotropic effects observed for specific CNVs such as those associated with *NRXN1*. In addition, a number of our subjects inherited *NRXN1* deletions from their parents. The detailed phenotype of these parents were not described in the medical records except in the family history, but the parents were ostensibly less affected than their children. This suggests that deletion in the *NRXN1* gene may not be fully penetrant, or interacts with other genes resulting in the variable phenotype. Further research efforts to investigate such variable phenotypes associated with this unstable genomic region will provide further insight into the role of *NRXN1* in the development of language delays, autism spectrum disorders, and physical features.

### Limitations

The accuracy and completeness of the clinical phenotype identified in this study is entirely dependent on the clinical information that was documented in the medical records of these subjects, often before the *NRXN1* deletions were identified in them. Because of the clinical variability exhibited in our cohort, the subjects were seen by a variety of specialists, which affected the completeness of data.

In addition, the parents were not formally assessed to ascertain their cognitive, physical, and behavioral phenotypes. As noted above, review of family history suggests that some parents may have shared similar phenotypes to their children. We are conducting further testing on both the subjects and their parents to better clarify developmental and/or social cognition issues in subjects and their parents.

For the deletion CNV significance test, we used the normal control data generated by different genomic profiling array platforms as reference. Knowing that the sensitivity and specificity differ from one array platform to another, this may not be an optimal comparison. However, the effort was made to minimize the detection bias between different array platforms. Here we have only chosen recent studies using array platform of similar resolution as ours. All these published articles reported the detection of smaller CNV, suggesting that technically all these array platforms were able to detect any CNV identified in this study. Thus this comparison, although an approximation, is on the conservative side.

Finally we acknowledge that while our clinically ascertained subjects were not drawn from a cohort with a single diagnosis such as autism or schizophrenia, they were ascertained from a heterogeneously affected group in whom genetic testing was considered clinically relevant. As a result, there is ascertainment bias and our findings may not reflect the true distribution of physical and developmental findings in the *NRXN1* deletion phenotype. Nevertheless, we have demonstrated that there are a number of other phenotypic features present in this clinical population beyond what has previously been identified in the literature.

## CONCLUSION

We found a wide range of phenotypic features in a group of subjects with *NRXN1* deletions who were clinically referred for genetic testing. These include subjects with autism spectrum disorders, mental retardation, language delays, hypotonia, hemangiomas, and the VACTERL association.
