# Supplementary material for: Deletions of NRXN1 (Neurexin-1) Predispose to a Wide Spectrum of Developmental Disorders
Source: Am J Med Genet B Neuropsychiatr Genet. 2010 Apr 7;153B(4):937–47. doi: 10.1002/ajmg.b.31063 (PMC3001124; doi:10.1002/ajmg.b.31063)
Supplement: Supplementary file 1 [file ajmg153B-0937-SD1.doc]

SUPPLEMENTARY MATERIAL

SUBJECT DESCRIPTIONS

Subject 1 had a 5 Mb deletion involving the entire *NRXN1* gene as well as the genes for *FSHR,* *LHCGR*, and *STN1*. The maternal study was normal and paternal studies could not be obtained. The subject presented with moderate mental retardation and a primary generalized epilepsy. He was well controlled on anti-epileptic medication and successfully weaned off medication at 15 years of age. His brain MRI was normal.

*FSHR* inactivating mutations have not been described to have significant effects in males [Thompson et al. 2008]. *LHCGR* mutations have been associated with Leydig cell hypoplasia [Kremer et al. 1995; Latronico et al. 1996; Laue et al. 1995]. There was no description of pubertal problems in our subject who was 16 years old at ascertainment. *STN1* is an ubiquitously expressed human homolog of *Stoned B,* a *Drosophila* gene that has been implicated in synaptic vesicle endocytosis [Martina et al. 2001]. However, this gene has not been shown to interfere with endocytosis *in vitro* [Martina et al. 2001]*.* To our knowledge, there are no other case reports in the literature of deletions in *STN1.*

Subject 2 had a 3.9 Mb de novo deletion. The subject presented with global developmental delays. Frontal bossing and a hemangioma on the neck were noted. He was noted to have failure to thrive. At his initial evaluation at our center, he was noted to have global developmental delays at the age of 2 years. Subsequently, he began receiving intensive alternative treatments for an autism spectrum disorder which were prescribed near his home; the origin of this diagnosis was not clear from the record.

Subject 3 had a 315 kb paternally inherited deletion of exons 1-5 and a part of intron 5. Her father was reportedly phenotypically normal. She presented with motor delays and hypotonia. Due to her young age there has been no developmental assessment to date.

Subject 4 had a 231 kb paternally inherited deletion of exons 1-5 and a part of intron 5. His father was reportedly phenotypically normal but felt awkward in social situations. He presented with pervasive developmental disorder, not otherwise specified. Hypotonia was noted on physical examination.

Subject 5 had a 139 kb de novo deletion. The deletion involved exons 3-5 and part of introns 2 and 5. She presented with VACTERL syndrome with a narrow aortic arch and ventricular septal defects, left polycystic kidney, imperforate anus, and a vaginal fistula. There was C1 dysraphism, 2-3 syndactyly of his toes, and a two vessel umbilical cord. She also had unilateral conductive hearing loss.

Subject 6 had a 257 kb deletion inherited from her mother. The deletion involved exons 1 and 2 and a part of intron 2. Her referring diagnoses were pervasive developmental disorder and developmental coordination disorder. There was a history of bilateral hip dysplasia and a history of a hemangioma. She was subsequently diagnosed with Attention Deficit-Hyperactivity Disorder. Treatment with stimulant medication led to emotional outbursts, and she has been treated with atomoxetine with good effect. She had a prolonged corrected QT interval of 457 ms on a screening electrocardiogram obtained while she was taking the osmotic-release formulation of methylphenidate. Her mother reportedly shared several phenotypic features with her.

Subject 7 had a 122 kb deletion. His mother does not share the deletion and paternal testing has not yet been performed. The deletion involved exons 1-3 and part of intron 3. He was referred for autism and moderate mental retardation. He had a conductive hearing loss that resolved with tympanostomy tubes. Adenoidectomy was performed for snoring. He was obese. Distinctive facial features were noted including slightly deep-set eyes. The ears were greater than 2 standard deviations above the mean length for his age.

Subject 8 had a 305 kb de novo deletion. The deletion involved exons 6-17 and partial deletions of introns 5 and 17. She had mild mental retardation and dysmorphic features, including a long face, malar hypoplasia, prominent tubular nose with pointed nasal tip, hypoplastic alae nase, long flat philtrum, thin vermilion, prominent chin, long slender fingers, and thin toes. The brain MRI was normal.

Subject 9 had a 154 kb deletion of unknown inheritance as she had been involved in child protective services. The deletion involved exons 6-8 and parts of introns 5 and 8. She was referred for language delay in the context of prenatal substance exposure. She had a low nasal bridge, small jaw, very smooth philtrum, slightly flat mid-face and prominent cheeks. A brain MRI was normal.

Subject 10 had a 139 kb de novo deletion involving intron 5 only. He had a diagnosis of Pervasive Developmental Disorder, not otherwise specified. He was noted to have a hemangioma on his back.

Subject 11 had a 75 kb maternally inherited deletion. The deletion involved intron 5 only. He was noted to have hypotonia and weakness. He had a right Poland anomaly.

Subject 12 had a 65 kb deletion of maternal inheritance. The deletion involved intron 5 only. She had a history of poor weight gain, frontal bossing, ventricular septal defect, atrial fenestration, and a patent ductus arteriosus. Further information on her clinical course was not available.

METHOD OF DELETION CONFIRMATION

A series of forward PCR primers were designed at about 2-3 Kb interval based on sequences between the last retaining array CGH probe before the deletion and the first deleted array CGH probe. One reverse PCR primer was designed based on the sequence near the first retaining array CGH probe after the deletion. Long-range PCR amplifications using the Platinum PCR SuperMix High Fidelity kit (Invitrogen Corp. Carlsbad, CA) were performed using the reverse primer and one of the forward primers. The shortest single PCR amplicon was sequenced afterwards to identify the exact sequence breakpoint.

Kremer H, Kraaij R, Toledo SP, Post M, Fridman JB, Hayashida CY, van Reen M, Milgrom E, Ropers HH, Mariman E and others. 1995. Male pseudohermaphroditism due to a homozygous missense mutation of the luteinizing hormone receptor gene. Nat Genet 9(2):160-4.

Latronico AC, Anasti J, Arnhold IJ, Rapaport R, Mendonca BB, Bloise W, Castro M, Tsigos C, Chrousos GP. 1996. Brief report: testicular and ovarian resistance to luteinizing hormone caused by inactivating mutations of the luteinizing hormone-receptor gene. N Engl J Med 334(8):507-12.

Laue L, Wu SM, Kudo M, Hsueh AJ, Cutler GB, Jr., Griffin JE, Wilson JD, Brain C, Berry AC, Grant DB and others. 1995. A nonsense mutation of the human luteinizing hormone receptor gene in Leydig cell hypoplasia. Hum Mol Genet 4(8):1429-33.

Martina JA, Bonangelino CJ, Aguilar RC, Bonifacino JS. 2001. Stonin 2: an adaptor-like protein that interacts with components of the endocytic machinery. J Cell Biol 153(5):1111-20.

Thompson MD, Percy ME, McIntyre Burnham W, Cole DE. 2008. G protein-coupled receptors disrupted in human genetic disease. Methods Mol Biol 448:109-37.
