# Supplementary material for: Deletions of NRXN1 (Neurexin-1) Predispose to a Wide Spectrum of Developmental Disorders
Source: Am J Med Genet B Neuropsychiatr Genet. 2010 Apr 7;153B(4):937–47. doi: 10.1002/ajmg.b.31063 (PMC3001124; doi:10.1002/ajmg.b.31063)
Supplement: Supplementary file 2 [file ajmg153B-0937-SD2.doc]

Table S-I: Deletion Confirmation Methods

| **Patient** | **Deletion Location (hg18 build)** | **Size of Deletion (Kb)** | **Confirmation Method** |
| --- | --- | --- | --- |
| 1 | 46,938,685-52,015,885 | 5,077 | FISH, Probe: RP11-800C7 |
| 2 | 50,128,256-54,050,713 | 3,923 | FISH, Probe: RP11-800C7 |
| 3 | 50,897,002-51,212,385 | 315 | PCR  Primers:  F:ACGTGGATTTTCAGCTCTGC R:GTGTGCCTTCTGAGGAAAGG |
| 4 | 50,936,914-51,167,934 | 231 | PCR  Primers  F: AAGTGCCAGGATTACCATGC R:TGCCAACTCACACCACAAAT |
| 5 | 50,920,082-51,059,469 | 139 | Not done |
| 6 | 51,059,410-51,316,396 | 257 | PCR  Primers:  F:TATGCCCATTTGGAAAGGTT R:TTTTGGAAGCTCCAAGCTGT |
| 7 | 51,090,504-51,212,385 | 122 | PCR  Primers:  F:TTCACATGGACAAAAATGCTG R:GGTAAATCGGCCCTGGTAAT |
| 8 | 50,522,892-50,827,767 | 305 | PCR  Primers:  F:CCATTCATCATGTGCAGATACA R:AATGCACTTTGCTATGCTCTGA |
| 9 | 50,689,280-50,853,329 | 164.0 | PCR  Primers:  F:GACTAGCCAAAAGCAAGGAGAA R:ATGCCCCATATTGTTATTCTCG |
| 10 | 50,714,297-50,853,329 | 139 | PCR  Primers:  F:TGGCCACTAGCTGTCACTAGAA R:TCATATTCAACTGGGAATGCTG |
| 11 | 50,735,499-50,811,018 | 76 | PCR  Primers:  F: TGCCAGACCTCTTGCTTTTT R:CAAAGCACTTTGAAAACATGAATC |
| 12 | 50,735,499-50,801,233 | 66 | PCR  Primers:F:TGCCAGACCTCTTGCTTTTT R:CAAAGCACTTTGAAAACATGAATC |
